# Dietary Approaches to Stop Hypertension (DASH) Diet, Incident Heart Failure and Its Associated Risk Factors in Australian Women

**DOI:** 10.3390/medicina62050985

**Published:** 2026-05-18

**Authors:** Lee Patricia Liao, Simone Marschner, Gary C. H. Gan, Liza Thomas, Allison Hodge, Haeri Min, Luigi Fontana, Sarah Zaman, Anushriya Pant

**Affiliations:** 1 Westmead Applied Research Centre, Faculty of Medicine and Health, University of Sydney, Sydney 2145, Australia; lee.liao@sydney.edu.au (L.P.L.); simone.marschner@sydney.edu.au (S.M.); liza.thomas@sydney.edu.au (L.T.); haeri.min@sydney.edu.au (H.M.);; 2 Department of Cardiology, Westmead Hospital, Sydney 2145, Australia; chgan84@gmail.com; 3 Department of Cardiology, Blacktown Hospital, Sydney 2148, Australia; 4 South West Clinical School, University of NSW, Sydney 2170, Australia; 5 Cancer Epidemiology Division, Cancer Council Victoria, Melbourne 3004, Australia; allison.hodge@cancervic.org.au; 6 Melbourne School of Population and Global Health, The University of Melbourne, Melbourne 3010, Australia; 7 Charles Perkins Centre, Faculty of Medicine and Health, University of Sydney, Sydney 2006, Australia; luigi.fontana@sydney.edu.au; 8 Department of Endocrinology, Royal Prince Alfred Hospital, Sydney 2050, Australia

**Keywords:** diet, heart failure, adherence, hypertension, diabetes mellitus, dietary intake

## Abstract

*Background and Objectives:* There is limited evidence supporting the incorporation of dietary patterns into heart failure (HF) management. The Dietary Approaches to Stop Hypertension (DASH) diet is linked to cardiovascular disease prevention, but evidence correlating DASH adherence to HF risk is sparse. This study is the first prospective investigation into the relationship between the DASH diet, incident HF and its associated risk factors—hypertension and diabetes mellitus (DM)—in Australian women. *Materials and Methods:* Survey data (2001–2022) from the Australian Longitudinal Study on Women’s Health (ALSWH) was analysed, where DASH diet scores were calculated from food frequency questionnaire (FFQ) responses and categorised into quintiles. Multivariable logistic regression was used to estimate odds ratios (ORs) and 95% confidence intervals (CIs) for the association between DASH adherence and incident HF. Cox proportional hazards models were used to calculate hazard ratios (HRs) and 95% CIs for secondary endpoints, hypertension and DM, and dietary exposure was modelled as a time-varying covariate. *Results:* 10 594 women (mean age 52.5 ± 1.45 years) participated and, at 21-year follow-up, there were 136 (1.3%) cases of HF, 2182 (20.6%) and 994 (5.7%) cases of hypertension and DM, respectively. After adjustment for covariates (including age and socioeconomic factors), no association was found between the highest DASH quintile and incident HF [OR 0.73, 95% CI: 0.37–1.43; *p* = 0.20]. However, adjusted HRs for hypertension and DM—0.73 (95% CI: 0.63–0.84; *p* < 0.001) and 0.65 (95% CI: 0.53–0.81; *p* < 0.001), respectively—indicated significant associations. *Conclusions:* In Australian women, DASH diet adherence was associated with a significantly lower risk of hypertension and DM, both of which are HF risk factors. The finding of no direct statistically significant association between the DASH diet and incident HF might reflect the small incidence of HF in our cohort.

## 1. Introduction

Heart Failure (HF) is the leading cause of hospitalisations [1] and disease burden, affecting 56 million individuals worldwide, including over 100,000 Australians [2,3]. In 2022, HF affected 49,900 Australian women over the age of 18 years, and while more men have HF compared to women (66% and 34% respectively), a higher number of women die from HF [4]. HF hospitalisation rates for women have risen by 41% for those aged 45–54 and by 18% for those aged 55–64 between 2015 and 2016 [5]. This figure is expected to rise due to an ageing population, presenting a dire need for novel preventative and treatment approaches.

There is increasing evidence that supports the benefits of dietary modification in cardiovascular disease (CVD) prevention [6,7], yet large clinical trials targeting patients with HF are lacking. Currently, sodium restriction is the only dietary recommendation incorporated into guidelines in HF patients [8], based on the potential benefits for fluid retention and hypertension prevention and treatment [9,10]. This has been met with controversy, partly due to the variations in recommended dietary intakes. In Australia, the HANDI (The Handbook of Non-Drug Interventions) project launched by the Royal Australian College of General Practitioners (RACGP) to promote non-drug related treatment approaches [11], rated the Dietary Approaches to Stop Hypertension (DASH) diet as the best diet for overall health and wellness [12].

The DASH diet is widely recommended for the management of hypertension, owing to its effect in lowering blood pressure [13]. Characterised by a low salt intake (no more than 2.5 g/day) [14], it emphasises high consumption of fruits, vegetables and plant based proteins from legumes and nuts, along with moderate intake of low-fat dairy products and limited consumption of red and processed meats and fats [15]. Beyond its anti-hypertensive effects, the diet is known for its anti-inflammatory and antioxidant properties. In individuals with untreated hypertension, it has been shown to decrease levels of cardiac troponin-1, a diagnostic marker of cardiac injury, and N-terminal pro B-type natriuretic peptide (NT-proBNP), a marker of heart stretch and volume overload [16,17]. Given that hypertension is a key risk factor for HF, the DASH diet could be beneficial for patients with HF; however, results from cohort studies have been conflicting, with some studies showing benefits of the DASH diet for HF outcomes [18,19] and others finding no association [20,21,22]. In one study, the DASH diet was also found to improve quality of life and exercise capacity in patients with HF [23]. Interestingly, Goyal et al. [19], found an inverse relationship between high adherence to the DASH diet and incident HF in those aged <75 years. Of note, in women with HF, greater adherence to the DASH diet, as opposed to the Mediterranean Diet, was found to be associated with lower mortality [24].

We therefore aimed to evaluate if high versus low adherence to the DASH diet was associated with HF incidence as well as HF risk factors, in Australian women.

## 2. Materials and Methods

The Australian Longitudinal Study on Women’s Health (ALSWH) is a prospective cohort study that began in 1996, enrolling over 41,000 women across Australia, with over 27 years of follow up [25]. Women were randomly selected from the national Australian Medicare Database across three age birth cohorts—born 1921–1926, 1946–1951, 1973–1978 with a fourth cohort (born 1989–1995) added more recently. Participants provided informed consent to regular surveys and data linkage to sources including hospital admissions, Medicare Benefits Schedule, Pharmaceutical Benefits Scheme, and the National Death Index. The methodology has been comprehensively outlined in prior publications [26].

For this analysis, women from the 1946–51 cohort, aged 45–50 years in 1996, were selected. The third survey, conducted in 2001, was used as the baseline as it was the first to collect dietary data. Participants were subsequently followed every 3 years until 2022. Inclusion criteria required completion of the 2001 survey and the availability of outcome data on incident HF (collected in surveys 9 and 10, in 2019 and 2022, respectively). Participants with implausible energy intake—defined as greater than twice that of the average estimated intake (~8700 kJ/day) [27,28], were excluded.

### 2.1. Dietary Assessment

Dietary intake was assessed from the third survey using responses from a validated 101—item food frequency questionnaire (FFQ) (Dietary Questionnaire for Epidemiological Studies version 2) [29]. This contained self-reported dietary intake where participants were asked to report their food and beverage consumption over 12 months (Supplementary Material Tables S1 and S2). Photographs of portion sizes were provided to participants indicate their usual portions.

### 2.2. DASH Diet

The DASH diet score is based on eight dietary components: fruits (4–5 servings/day), vegetables (4–5 servings/day), nuts and legumes (4–5 servings/week), low-fat dairy (2–4 servings/day), whole grains (6–8 servings a day), sodium (<2.5 g/day [14]), sweetened beverages (5 or fewer/week), and red and processed meat (2 or fewer servings/day). Recommended intakes have been taken from the RACGP diet plan [12]. Foods included in each category are shown in Supplementary Material Table S3. The DASH diet score, proposed by Fung et al., [30] was used for the current study, with participants categorised into quintiles according to the amount of intake of the specific item and scored 1–5 points depending on the quintile. Scores were then totalled and the DASH score obtained. Overall, DASH scores were categorised into quintiles.

### 2.3. Primary and Secondary Follow-Up and End Points

The primary endpoint was incident HF. This was identified through self-report on follow-up surveys 9 and 10. Participants were asked to respond ‘yes’ or ‘no’ to the question: “In the past three years, have you been diagnosed or treated for congestive heart failure?” Secondary endpoints were incident hypertension and diabetes mellitus (DM), both of which were also self- reported. These conditions were assessed in follow-up surveys conducted every 3 years until 2022 and defined as the first-reported diagnosis among patients without the condition at baseline. At baseline, the presence of DM was determined by a response of “Yes” to either of the following questions: “In the past three years, have you been diagnosed or treated for: Insulin dependent (type 1) diabetes?” and “In the past three years, have you been diagnosed or treated for: non-insulin dependent (type II) diabetes?”. For follow-up surveys, diabetes was identified through the same questions in surveys 9 and 10, and DM by a response of “Yes” to the question: “In the past three years have you been diagnosed with or treated for: Diabetes (high blood sugar)?” in surveys 4 to 8. Hypertension was assessed consistently across all surveys based on a “Yes” response to the question: “In the past three years, have you been diagnosed or treated for: High blood pressure (hypertension)”.

### 2.4. Baseline Assessment and Assessment of Covariates

Based on the current literature [15,31,32,33,34], the following variables were chosen for baseline characteristics. Continuous variables selected were age and area of residence determined by Accessibility and Remoteness Index of Australia (ARIA+). Categorical variables were body mass index (BMI), country of birth, highest qualification attained and main occupation. The presence of medical comorbidities (hypertension and DM) and lifestyle factors (physical activity, smoking and alcohol status) were also included. A description of how these were measured can be found in Supplementary Material Table S2.

### 2.5. Statistical Analysis

In the primary analysis, to evaluate the association of the DASH diet with incident HF, multivariable logistic regression was used to estimate the adjusted odds ratio (aOR) and corresponding 95% confidence intervals (CIs). Adjustments were made for age, area of residence, country of birth, highest qualification level and main occupation (model 1) and obesity, a prior diagnosis of hypertension and DM (model 2). As such model 2 estimates the direct effect of diet on incident HF independent of these mediators. HF was first assessed in surveys 9 and 10 and not in earlier waves, and the exact date of HF onset was unknown, so a time-to-event analysis was not performed. For the secondary outcomes of DM and hypertension, those with a prior diagnosis of these secondary outcomes at baseline were not included in the analysis. Cox proportional hazards models were used to estimate the adjusted hazard ratio (aHR) and corresponding 95% CI for the association between the DASH diet and each secondary outcome.

The DASH diet adherence was assessed at two time points, surveys 3 and 7, and hence put in the model as a time varying covariate. The most recent measure was carried forward for subsequent follow-up waves and because diet was not reassessed thereafter, changes in dietary patterns over the remaining follow-up period could not be captured.

Adjustments were made for age, area of residence, highest qualification attained, main occupation, country of birth and prior diagnosis of hypertension for the secondary outcome of DM and prior DM diagnosis for the secondary outcome of hypertension. Statistical significance was determined by a *p* < 0.05. All analyses were conducted using R version 4.3.1 (R Foundation for Statistical Computing, Vienna, Austria) within the RStudio environment (version 2025.06.13).

## 3. Results

### 3.1. Study Sample

**Figure 1:**
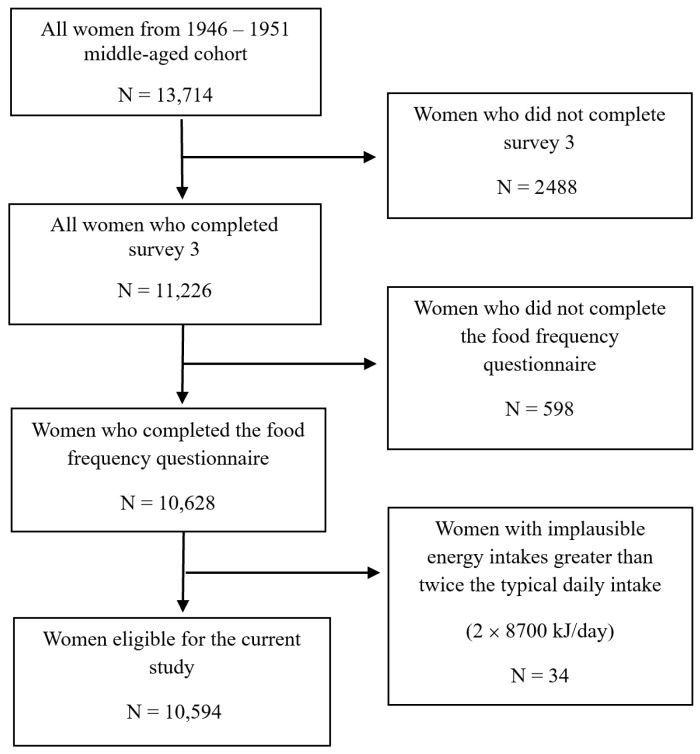
Flow chart showing eligibility criteria for this study.

ALSWH recruited 13,714 women into the 1946–51 cohort. For the current study, for women who did not complete survey 3 (n = 2488), the FFQ at survey 3 (n = 598), or reported implausible energy intakes (n = 34), were excluded. The final study cohort included 10,594 women (Figure 1).

### 3.2. Baseline Characteristics

Participants had a mean age of 52.5 ± 1.5 years and mean BMI of 26.8 kg/m^2^. The median DASH score was 25.0 (maximum score of 40) and 17.7% of women were in the highest adherence group (quintile 5) and 20.2% in the lowest adherence group (quintile 1). Women with higher adherence to the DASH diet had healthier lifestyle practices than those with lower adherence, including higher levels of physical activity and lower incidence of smoking and high-risk drinking (Table 1). Compared to those in lower quintiles, women in quintile 5 showed a lower proportion who were obese (n = 326, 18%) and a higher proportion who held a tertiary education qualification (n = 529, 28%). Among known risk factors for HF, hypertension was most prevalent in quintile 2 (n = 744, 30%) and least common in quintile 5 (n = 465, 25%). There was no significant difference in incidence of DM observed between the quintiles.

**Figure 2:**
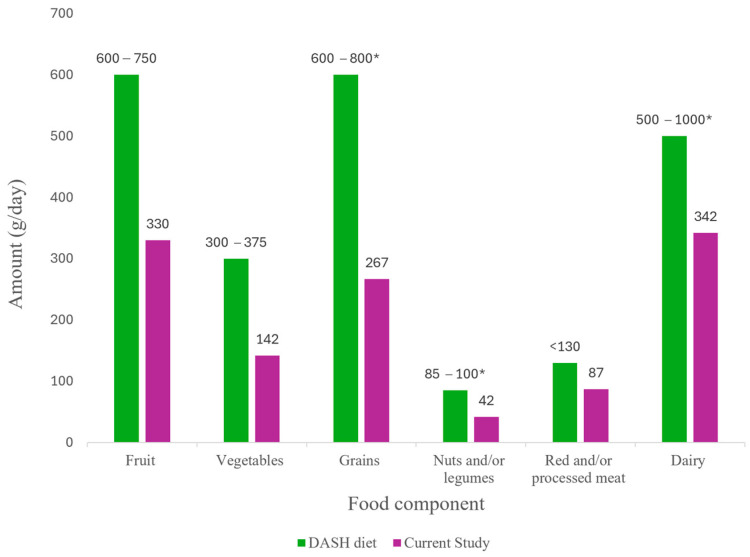
Individual food component intakes for participants in the current study in quintile 5 compared to the DASH diet recommendations for main food groups. Recommended intakes are from the RACGP DASH diet plan [12], and serving sizes are converted to g/day as per Australian Dietary Guidelines [35]. Intakes for each quintile are shown in Supplementary Material Table S4. Note that the DASH diet recommends low fat-dairy but there was no differentiation in the data in the current study. * denotes the recommended range for one type of food in that category.

Figure 2 shows the individual food component intakes for the participants compared with DASH diet recommendations. Intakes for all quintiles can be found in Supplementary Material Table S4. All women consumed less than the DASH diet’s recommended daily intake of sodium (<2500 mg/day) [14], although it is important to note that estimates of sodium intake from the FFQ are based on reported consumption of high-sodium foods, rather than direct measurement of total sodium intake. Red and/or processed meat was within the recommended DASH diet daily intake of less than 130 g/day. Intake of vegetables and fruits for all quintiles did not meet the DASH diet requirements, with women in quintile 5 consuming approximately 50% less than recommended amounts (Figure 2).

### 3.3. Primary Outcome: Association Between DASH Diet and Incident HF

After 21 years of follow-up there were 136 (1.3%) HF cases. In both the unadjusted and multivariable models, there was no statistically significant association between the DASH diet adherence and incident HF [Quintile 5: aOR 0.73; 95% CI 0.37, 1.43; *p* = 0.60] (Table 2).

### 3.4. Secondary Outcome: Association Between DASH Diet and Risk of Hypertension and DM

There were 2182 (20.6%) cases of hypertension and 994 (9.4%) cases of DM at 21 year follow up. Multivariable analysis showed a significantly lower risk of hypertension and diabetes with high versus low adherence to the DASH diet (*p* < 0.001) (Table 3). Table 3 shows the multivariable adjusted hypertension and DM models.

## 4. Discussion

This is the first prospective study in an Australian population of women to investigate the relationship between adherence to the DASH, incidence of HF, and its associated risk factors. In this large, prospective cohort, women with the highest adherence to the DASH diet had a 27% lower risk of hypertension and a 35% lower risk of DM, compared to women with the lowest DASH diet adherence. However, despite these significant reductions in key HF risk factors, no statistically significant association was found between the DASH diet and HF, likely due to low HF incidence in this relatively young and healthy population of women.

To date, research into the association of the DASH diet on HF outcomes has been limited, with existing studies producing conflicting results. A lower HF risk was demonstrated by a Swedish cohort study (n = 76,222) where highest adherence to the DASH diet was associated with a lower hazard for incident HF (HR 0.85, 95% CI 0.77, 0.95) [18]. Similarly, a higher risk was observed in lower adherence groups in the Reasons for Geographic And Racial Differences in Stroke (REGARDS) cohort (n = 30,239) for those less than 74 years [19] [HR 0.60, 95% CI 0.41,0.86], but no association for those older. In contrast, a lack of association was found in the Cardiovascular Health Study and Multi-ethnic Study of Atherosclerosis; although, the latter observed a lower risk with high dietary adherence for those younger than 75 years [20,21]. Both these cohort studies (n = 4490 and n = 4478 respectively) had a smaller sample size than the Swedish and REGARDS studies. It follows that smaller cohort (n = 10,594) and low number of HF cases (1.3%) accounts for the under-ascertainment of associations and bias towards the null.

Additionally, variations in study design and population characteristics—including sex distribution and follow-up duration—may account for inconsistencies across studies. For instance, the shorter follow-up periods in the Swedish and REGARDS cohorts may have resulted in lower attrition and more HF events, thereby increasing statistical power. Differences in DASH diet scoring across studies can shape the clarity of observed associations. Cut-off scores used in some scoring methods [36], can compress participants into low-adherence categories, whereas the Fung et al. [30], score used in this study, retains population-level dietary variation retaining meaningful variation gradients.

Furthermore, previous study populations found variability by age group, such that the association between DASH and HF risk was more pronounced in participants under 75 [19,21]. This is in contrast to our participants, given the age range at final follow up was 71–77 years.

Moreover, while the Swedish and REGARDS cohorts included participants of both sexes, the present study focused exclusively on women—a population historically underrepresented in HF clinical trials [37]. Interestingly, in the Swedish mammography observational study in women (n = 36,019), those in the highest adherence group had a 37% lower rate of HF incidence [38]. However, this cohort was 70 years and older which may explain the stronger association.

It is important to note that our population appeared to consist of relatively healthy women with low smoking rates, reasonable BMI distribution and a generally favourable lifestyle profile. In addition, the cohort was relatively young (aged 45–50 years at baseline). These factors may explain the attenuated association between DASH diet adherence and HF risk. Furthermore, certain components of participants’ baseline diet, namely sodium and red and processed meat consumption, were already low and consistent with DASH guidelines. The association of red meat and HF has previously been established, with increased consumption correlated with an increase in HF risk [39]. In fact, Ibsen and colleagues [18], showed that substituting red and processed meats with fruits, vegetables, legumes, low-fat dairy and whole grains lowered risk of HF by 8–12%.

As mentioned earlier, the majority of participants in the current study had favourable lifestyle habits and were physically active. Del Gobbo et al. [20], found that the presence of ≥4 healthy lifestyle habits resulted in a 45% risk reduction in developing HF. These findings suggest that overall lifestyle habits may play a more significant role in shaping HF risk than dietary choices alone. Interestingly, a close examination of baseline data from the Swedish Mammography Cohort [38] (a subset of the Swedish cohort), revealed that older women with less desirable lifestyle habits (higher BMI, higher cholesterol levels and current smokers) were at greater risk of developing HF. This finding reinforces the interpretation that the low incidence of HF in the current study may be attributable to the cohort’s overall healthy lifestyle profile.

Increased adherence to the DASH diet was associated with lower hypertension risk in this study, reinforcing its recognised effectiveness in treating hypertension, a key modifiable contributor to HF [15,33]. We demonstrated a progressively higher risk of hypertension in women with lower DASH diet adherence. This inverse relationship was also found by Folsom et al. [40], who studied a population of women aged 55 to 69 years (n = 41,386) over 12 years. The authors found a 13% risk reduction, after adjusting for age and energy intake, by those in quintile 5 compared to quintile 1. However, since few women achieved high adherence to the diet, it was concluded that perhaps very high adherence is required to achieve beneficial effects from the diet, raising the possibility that substantial compliance is necessary to elicit the intended health benefits of the diet.

Higher adherence to the DASH diet was also associated with a decreased risk of developing DM. This is consistent with the meta-analysis of randomised controlled trials (n = 1239) on the effect of the DASH diet on glycaemic control, where the diet was shown to improve insulin sensitivity [41]. Even a short-term study of 1 year showed that the DASH diet had a protective effect against Type 2 DM [42]. Although Type 2 DM specifically is the known risk factor for HF [34], the survey question on diabetes between 2004 and 2016 did not distinguish between Type 1 and Type 2 DM. Consequently, the observed association between DASH diet adherence and DM cannot be attributed to a specific diabetes type within our analysis. However, given the age of women in this cohort is beyond the typical age for Type 1 DM to have manifested [43], it is likely that the vast majority of “new” cases of DM were Type 2.

### Limitations

Compared with other cohort studies, the number of incident HF cases in the current study was small (n = 136), likely due to the relatively healthy cohort of women who had favourable lifestyle factors; this may have contributed to the lack of statistical significance. HF was assessed only at two time points in 2019 and 2022. It is likely that some participants diagnosed with HF between 2001 and 2016 were missed or had died and hence may not have reported it then. This is likely as incidence of HF increases markedly beyond the age of 60, with most new diagnoses in Australia going to individuals aged 65 years and above [44]. Furthermore, there is potential for reverse causality whereby patients at risk of HF may adopt a specific dietary pattern, e.g., a DASH diet. While multivariable adjustment was carried out, residual confounding and reverse causation remain possible.

The occurrence of HF, DM and hypertension was determined by self-report, which can lead to inaccuracies [45]. However, a study found that self-reporting of diabetes and hypertension was relatively reliable; however, less so for HF, with under reporting possibly due to the often relatively asymptomatic and fluctuating nature of the disease [46]. Underestimation of energy intake particularly food with higher energy densities, and inaccurate estimations of protein intake is well-documented [47,48]. In the current study, diet was assessed through FFQ, a validated measurement of food intake [49,50]. However, the self-reported nature of the FFQ may have led to over or under-reporting of dietary data and resulted in potential recall bias. In particular, the relatively low sodium intake reported in this study, may reflect limitations inherent to the FFQ methodology which relies on predefined high-sodium foods. Consequently, actual intake may be underestimated due to unaccounted sources, therefore influencing the association between diet and outcomes. Furthermore, the assessment of diet at only two time points when dietary patterns over 20+ years may change might introduce potential misclassification.

## 5. Conclusions

High adherence to the DASH diet was associated with a reduced risk of developing hypertension and diabetes mellitus, both risk factors for HF, in Australian women. The association between the DASH diet and HF incidence was statistically insignificant, although this may be attributed to the small number of HF events in a relatively healthy population. Future studies could investigate the correlation between individual DASH dietary components and HF. Furthermore, the influence of DASH diet on specific HF subgroups, such as a more significant effect in HF with preserved or reduced ejection fraction, would give further insights into the benefit of the diet in this population of patients.

## Figures and Tables

**Table 1:** Characteristics of participants age 45–50 from the Australian Longitudinal Study on Women’s Health (1946–51 cohort) according to quintiles of Dietary Approaches to Stop Hypertension diet score adherence.

Characteristics	Quintile 1 11–21 Points (n = 2141) n (%)	Quintile 2 22–24 Points (n = 2446) n (%)	Quintile 3 25–26 Points (n = 1852) n (%)	Quintile 4 27–29 Points (n = 2284) n (%)	Quintile 5 30–39 Points (n = 1871) n (%)	Overall (n = 10,594) n (%)	*p*-Value ^1^
**DASH score (points)**	19.2	23.1	25.5	27.9	31.8	25.0 *	<0.001
**Mean Age (years)**	52.4 ± 1.5	52.5 ± 1.5	52.5 ± 1.5	52.6 ± 1.5	52.7 ± 1.4	52.5 ± 1.5	0.003
**BMI (kg/m^2^)**							<0.001
Underweight (<18.5)	35 (1.6)	26 (1.1)	35 (1.9)	21 (0.9)	26 (1.4)	143 (1.4)	<0.001
Healthy (18.5–25)	818 (39)	1011 (42)	721 (39)	1005 (44)	911 (49)	4466 (42)	
Overweight (25–30)	674 (32)	813 (33)	605 (33)	741 (33)	596 (32)	3429 (33)	
Obese (>30)	597 (28)	583 (24)	481 (26)	501 (22)	326 (18)	2488 (24)	
Country of Birth **							<0.001
Australian-born	1723 (81)	1888 (78)	1423 (78)	1719 (76)	1372 (74)	8125 (78)	
Other English-speaking	242 (11)	302 (13)	249 (14)	336 (15)	309 (17)	1438 (14)	
Europe	111 (5.2)	147 (6.1)	108 (5.9)	135 (6.0)	108 (5.8)	609 (5.8)	
Asia	27 (1.3)	51 (2.1)	40 (2.2)	58 (2.6)	49 (2.6)	225 (2.1)	
Other	13 (0.6)	21 (0.9)	14 (0.8)	16 (0.7)	20 (1.1)	84 (0.8)	
**Residential Area**							<0.001
Major cities	620 (29)	844 (35)	643 (35)	833 (36)	659 (35)	3599 (34)	
Inner regional	919 (43)	988 (40)	746 (40)	914 (40)	768 (41)	4335 (41)	
Outer regional	498 (23)	497 (20)	384 (21)	452 (20)	366 (20)	2197 (21)	
Remote/Very remote	104 (4.9)	117 (4.8)	79 (4.3)	85 (3.7)	78 (4.2)	463 (4.4)	
**Highest Qualification**							<0.001
Year 12 or below	1490 (70)	1506 (62)	1083 (59)	1245 (55)	819 (44)	6143 (58)	
Certificate/diploma	394 (18)	563 (23)	408 (22)	532 (23)	517 (28)	2414 (23)	
University	254 (12)	368 (15)	360 (19)	505 (22)	529 (28)	2016 (19)	
**Main occupation**							<0.001
Manager	161 (8.4)	189 (8.6)	137 (8.3)	163 (7.9)	147 (8.8)	797 (8.4)	
Professional/Associate Professional	401 (21)	545 (25)	472 (29)	660 (32)	624 (37)	2702 (29)	
Trade/Labourer/Transport	314 (16)	293 (13)	183 (11)	234 (11)	141 (8.4)	1165 (12)	
Clerical/Sales/Service	438 (23)	514 (24)	411 (25)	475 (23)	374 (22)	2212 (23)	
Unpaid/No work	593 (31)	645 (30)	449 (27)	529 (26)	385 (23)	22,601 (27)	
**Physical Activity Level**							<0.001
Sedentary	547 (27)	480 (20)	287 (16)	283 (13)	154 (8.5)	1751 (17)	
Low	711 (35)	831 (35)	636 (36)	708 (32)	501 (28)	3387 (33)	
Moderate	325 (16)	485 (21)	379 (21)	526 (24)	448 (25)	2163 (21)	
High	442 (22)	551 (23)	473 (27)	680 (31)	702 (39)	2848 (28)	
**Smoking status**							<0.001
Never	1112 (52)	1452 (59)	1228 (66)	1461 (64)	1217 (65)	6470 (61)	
Ex-smoker	454 (21)	600 (25)	410 (22)	591 (26)	539 (29)	2594 (24)	
Smoker	575 (27)	393 (16)	214 (12)	232 (10)	115 (6.1)	1529 (14)	
**Alcohol**							<0.001
Non-drinker/Rarely	982 (46)	967 (40)	754 (41)	879 (39)	756 (40)	4338 (41)	
Low risk drinker	1013 (47)	1305 (53)	1003 (54)	1283 (56)	1042 (53)	5646	
High risk/Risky drinker	142 (6.6)	170 (7.0)	92 (5.0)	121 (5.3)	71 (3.8)	596 (5.6)	
**Hypertension**	663 (31)	744 (30)	540 (29)	633 (28)	465 (25)	3045 (29)	<0.001
**Diabetes**	84 (3.9)	126 (5.2)	108 (5.8)	120 (5.3)	94 (5.0)	532 (5.0)	0.081

^1^ Kruskal–Wallis rank sum test; Pearson’s Chi-squared test; * median DASH score; ** measured only at survey 1.

**Table 2:** Odds ratios and 95% confidence intervals for association of DASH diet and HF incidence.

	DASH Diet Score					
	Q1	Q2	Q3	Q4	Q5	*p*-Value
**Range**	11–21	22–24	25–26	27–29	30–39	
**Cases**	25	27	30	33	21	
**OR (95% CI)**						
Unadjusted	1.0 (ref)	0.86 (0.5, 1.5)	1.21 (0.71, 2.09)	1.07 (0.64, 1.83)	0.80 (0.44, 1.43)	0.60
Age and socioeconomic Status * adjusted	1.0 (ref)	0.96 (0.54, 1.75)	1.21 (0.67, 2.21)	1.14 (0.64, 2.04)	0.72 (0.36, 1.41)	0.20
Comorbidities ** adjusted	1.0 (ref)	0.85 (0.49, 1.48)	1.19 (0.70, 2.06)	1.08 (0.64, 1.84)	0.83 (0.46, 1.49)	0.60
Multivariable # adjusted	1.0 (ref)	0.94 (0.52, 1.72)	1.18 (0.65, 2.16)	1.11 (0.63, 2.00)	0.73 (0.37, 1.43)	0.20

Abbreviations: CI—Confidence Interval; OR—Odds Ratio; ref—reference; Q—Quintile; Quintile 1 (Q1) is the reference point; * age and socioeconomic status: age, residence, country of birth, highest qualification attained, occupation; ** comorbidities: diabetes mellitus, hypertension; # multivariable: age, residence, county of birth, highest qualification attained, occupation, obesity, diabetes mellitus, hypertension.

**Table 3:** Hazard ratios and 95% confidence intervals for association of DASH diet and secondary outcomes of hypertension and diabetes mellitus.

	DASH Diet Score					
	Q1	Q2	Q3	Q4	Q5	*p*-Value
**Range**	11–21	22–24	25–26	27–29	30–39	
**Hypertension**						
**HR (95% CI)**						
Unadjusted	1.0 (ref)	0.94 (0.83, 1.06)	0.93 (0.82, 1.05)	0.80 (0.70, 0.90)	0.72 (0.63, 0.83)	<0.001
Multivariable * adjusted	1.0 (ref)	0.95 (0.84, 1.08)	0.93 (0.82, 1.06)	0.80 (0.70, 0.92)	0.73 (0.64, 0.84)	<0.001
**Diabetes Mellitus** **HR (95% CI)**						
Unadjusted	1.0 (ref)	0.81 (0.68, 0.96)	0.85 (0.71, 1.00)	0.66 (0.55, 0.79)	0.55 (0.45, 0.67)	<0.001
Multivariable * adjusted	1.0 (ref)	0.83 (0.69, 1.00)	0.87 (0.73, 1.05)	0.72 (0.59, 0.87)	0.65 (0.53, 0.81)	<0.001

* Multivariable: age, residence, county of birth, highest qualification attained, occupation, obesity, diabetes mellitus, hypertension.

## Data Availability

Data from the Australian Longitudinal Study on Women’s Health (ALSWH) are not publicly available. Researchers may apply for access through the ALSWH data access process (www.alswh.org.au) subject to approval and data use agreements.

## Supplementary Materials

The following supporting information can be downloaded at: https://www.mdpi.com/article/10.3390/medicina62050985/s1, Table S1: Questions and response options from ALSWH used for baseline characteristics; Table S2: Questions and response options to determine typical eating habits and patterns for calculation of DASH scores. Table S3: Foods included as part of the eight dietary components of the DASH diet. Table S4. Individual food component intakes for participants in the current study compared to DASH diet recommendations. DASH scores are shown for each quintile.

## Abbreviations

The following abbreviations are used in this manuscript:

ARIA+	Accessibility and Remoteness Index of Australia
aHR	Adjusted Hazard Ratio
aOR	Adjusted Odds Ratios
ALSWH	Australian Longitudinal Study on Women’s Health
BMI	Body Mass Index
CVD	Cardiovascular Disease
CIs	Confidence Intervals
DM	Diabetes Mellitus
DASH	Dietary Approaches to Stop Hypertension
FFQ	Food Frequency Questionnaire
HR	Hazard Ratios
HF	Heart Failure
NT-proBNP	N-terminal pro B-type natriuretic peptide
OR	Odds Ratios
RACGP	Royal Australian College of General Practitioners
HANDI	The Handbook of Non-Drug Interventions
