# Supplementary material for: Dietary Approaches to Stop Hypertension (DASH) Diet, Incident Heart Failure and Its Associated Risk Factors in Australian Women
Source: Medicina (Kaunas). 2026 May 18;62(5):985. doi: 10.3390/medicina62050985 (PMC13208208; doi:10.3390/medicina62050985)
Supplement: Supplementary file 1 [file medicina-62-00985-s001.zip › medicina-4294569-supplementary.pdf]

Supplementary S1

Table S1. Questions and response options from ALSWH used for baseline characteristics

Variable	Method of Determination	Options	Categorisation
BMI	<u>Question:</u><br>How much do you weigh?<br>How tall are you?<br><br>Calculation<br>(Weight(kg)/(Height(m))^2)<br><br>Grouped according to WHO BMI group category	______kg _____cm	Underweight<br>Healthy<br>Overweight<br>Obese
Country of Birth	<u>Question:</u><br>In which country were you born? (Circle one number only)	1. Australia<br>2. United Kingdom<br>3. Italy<br>4. Greece<br>5. New Zealand<br>6. Vietnam<br>7. Other (Please specify on line)	Australia born<br>Other-English speaking<br>Europe<br>Asia
Residential Area	<u>Question:</u><br>What is your residential postcode?<br><br>These are then linked to ARIA+ scores and classified according to remoteness	___ ___ ___ ___<br><br>Major cities, Inner regional, Outer regional, remote and very remote	Major cities<br>Inner regional<br>Outer regional<br>Remote
Highest Qualification	<u>Question:</u><br>What is the highest qualification you have completed?	1. No formal qualifications<br>2. School or Intermediate Certificate (or equivalent)<br>3. Higher School or Leaving Certificate (or equivalent)<br>4. Trade/apprenticeship (eg Hairdresser, Chef)<br>5. Certificate/diploma (eg Child Care, Technician)<br>6. University degree<br>7. High University degree (eg Grad Dip, Masters, PhD)	Year 12 or below<br>Certificate/Diploma<br>University

Main Occupation	<u>Question:</u><br>We would like to your and your partner's main occupation now	1. Manager<br>2. Professional<br>3. Associate Professional<br>4. Tradesperson or related worker<br>5. Advanced clerical or service worker<br>6. Intermediate clerical, sales or service worker<br>7. Intermediate production or transport worker<br>8. Elementary clerical, sales or service worker<br>9. Labourer or related worker<br>10. No paid job<br>11. Don't know	Manager<br>Professional/Associate Professional<br>Trade/Labourer/Transport<br>Clerical/Sales/Service<br>Unpaid/No work
Physical Activity Level	<u>Question</u>:<br>If you add up all the times you spent in each activity last week, how much time did you spend altogether doing each type of activity? and duration of walking, moderate, and vigorous exercise?<br><br>Responses were converted into weekly energy expenditure using metabolic equivalent task (MET) value. Participants were then grouped into four physical activity categories based on weekly energy expenditure.	1. Walking briskly (for recreation or exercise, or to get from place to place) ______times<br>2. Moderate leisure activity (like social tennis, moderate exercise classes, recreational swimming, dancing) ______times<br>3. Vigorous leisure activity (that makes you breathe harder or puff and pant like aerobics, competitive sport, vigorous cycling, running, swimming) ______times<br>4. Vigorous household or garden chores (that make you breathe harder of puff and pant) ______times	Sedentary<br>Low<br>Medium<br>High
Smoking Status	<u>Question:</u><br>How often do you currently smoke cigarettes or any tobacco products?<br><br>On average, how many cigarettes do you smoke each week?<br><br>At what age did you finally stop smoking daily?<br><br>Combinations of responses to the relevant survey items were allocated to each category for smoking status.	1. Daily<br>2. At least weekly (but not daily)<br>3. Less often than weekly<br>4. Not at all<br><br>__<br>_______ cigarettes/week<br><br>I have never smoked daily<br>OR<br>I stopped smoking daily at _____ years old	Never<br>Ex-smoker<br>Smoker

Alcohol	<u>Question:</u><br>How often do you usually drink alcohol?	1. I never drink alcohol<br>2. I drink rarely<br>3. Less than once a month<br>4. Less than once a week<br>5. On 1 or 2 days a week<br>6. On 3 or 4 days a week<br>7. On 5 or 6 days a week<br>8. Every day	Non-drinker/Rarely<br>Low risk drinker<br>High risk/risky drinker
	On a day when you drink alcohol, how many standard drinks do you usually have?	1. I don't drink alcohol<br>2. Non-drinker<br>3. 1 or 2 drinks per day<br>4. 3 or 4 drinks per day<br>5. 5 to 8 drinks per day<br>6. 9 or more drinks per day	
	How often do you have five or more standard drinks of alcohol on one occasion?	1. Never<br>2. Less than once a month<br>3. About once a month<br>4. About once a week<br>5. More than once a week<br>6. Non-drinker	
	Based on NHMRC guidelines, a variable for alcohol consumption status was derived from the frequency and quantity items.		
Hypertension	<u>Question:</u><br>In the past three years, have you been diagnosed or treated for:<br>High blood pressure (hypertension)	Yes or No	

Diabetes	Question:<br>Have you ever been told by a doctor that you have:<br>Diabetes (high blood sugar)	Yes or No
	Have you ever been told by a doctor that you have?<br>Insulin dependent (type 1) diabetes<br>Have you ever been told by a doctor that you have?<br>Non-insulin dependent (type 2) diabetes	1. Never<br>2. Yes, in the last 2 years<br>3. Yes, more than 2 years ago<br>4. both

Table S2. Questions and response options to determine typical eating habits and patterns for calculation of DASH scores.

Food component	Method of Determination	Options
Fruits	Question:<br>How many pieces of fresh fruit do you usually eat per day? (Count half a cup of diced fruit, berries or grapes as ½ piece)	1. I don't eat fruit<br>2. Less than 1 piece of fruit/day<br>3. 1 piece of fruit/day<br>4. 2 pieces of fruit/day<br>5. 3 pieces of fruit/day<br>6. 4 or more pieces of fruit/day
Vegetables	Question:<br>How many different vegetables do you eat per day? (Count all types, fresh, frozen or tinned)	1. Less than 1 vegetable/day<br>2. 1 vegetable/day<br>3. 2 vegetables/day<br>4. 3 vegetables/day<br>5. 4 vegetables/day<br>6. 5 vegetables/day<br>7. 6 or more vegetables/day
Dairy	Question:<br>What type of milk do you usually use?	1. None<br>2. Full cream milk<br>3. Reduced fat milk<br>4. Skim milk<br>5. Soya milk
	Question:<br>How much milk do you usually use per day? (Include flavoured milk and milk added to tea, coffee etc).	1. None<br>2. Less than 250 mL<br>3. Between 250 and 500 mL<br>4. Between 500 and 750 mL<br>5. 750 mL or more
	Question:<br>What types of cheese do you usually eat?	1. I don't eat cheese<br>2. Hard cheeses (e.g. parmesan, romano)<br>3. Firm cheeses (e.g. cheddar, edam)<br>4. Soft cheeses (e.g. camembert, brie)<br>5. Ricotta or cottage cheese<br>6. Cream cheese<br>7. Low fat cheese

Bread	Question:<br>What type of bread do you usually eat?	1. I don't eat bread<br>2. White bread<br>3. Wholemeal bread<br>4. Multigrain bread<br>5. Rye bread<br>6. Other (please specify):
Fats/Oils	Question:<br>What type of spread do you usually use on bread or toast?	1. I don't use spread<br>2. Butter<br>3. Margarine<br>4. Low fat margarine<br>5. Other (please specify):
	Question:<br>What type of fat or oil do you usually use for cooking?	1. I don't use fat or oil<br>2. Butter<br>3. Margarine<br>4. Vegetable oil<br>5. Olive oil<br>6. Other (please specify):
	Question:<br>What type of fat or oil do you usually use in salads or on vegetables?	1. I don't use fat or oil<br>2. Vegetable oil<br>3. Olive oil<br>4. Other (please specify): _
Sugar	Question:<br>On average, how many teaspoons of sugar or sweetener do you use each day?	1. None<br>2. 1 – 4 tspn/day<br>3. 5 – 8 tspn/day<br>4. 9 – 12 tspn/day<br>5. More than 12 tspn/day
Eggs	Question:<br>On average, how many eggs do you eat each week?	1. I don't eat eggs<br>2. < 1 egg/week<br>3. 1 – 2 eggs/week<br>4. 3 – 5 eggs/week<br>5. More than 6 eggs/week
Cereal, Foods, Sweets & Snacks	Question:<br>Over the LAST 12 MONTHS, on average, how often did you eat the following foods?<br>☐ All Bran<br>☐ Sultana Bran™, FibrePlus™, Branflakes™<br>☐ Weet Bix™, Vita Brits™, Weeties™<br>☐ Cornflakes, NutriGrain™, Special K™<br>☐ Porridge<br>☐ Muesli<br>☐ Rice<br>☐ Pasta or noodles (include lasagne)<br>☐ Crackers, crispbreads, dry biscuits<br>☐ Sweet biscuits or cookies	1. Never or less than once per month<br>2. 1–3 times per month<br>3. Once per week<br>4. 2–4 times per week<br>5. 5–6 times per week<br>6. Once per da<br>7. 2 or more times per day

	☐ Cakes, sweet pies, tarts, pastries, sponge cake<br>☐ Pizza<br>☐ Hamburger with a bun<br>☐ Chocolate<br>☐ Flavoured milk drink (cocoa, Milo™ etc)<br>☐ Custard<br>☐ Fruit juice (bottled or from concentrate)<br>☐ Jam, marmalade, honey or syrups<br>☐ Vegemite™, Marmite™ or Promite™	
Dairy products, meat and fish	<u>Question:</u><br>Over the LAST 12 MONTHS, on average, how often did you eat the following foods?<br><br>☐ Cheese<br>☐ Ice-cream<br>☐ Yoghurt<br>☐ Beef<br>☐ Veal<br>☐ Chicken<br>☐ Lamb<br>☐ Pork<br>☐ Bacon<br>☐ Ham<br>☐ Corned beef, luncheon meats or salami<br>☐ Sausages or frankfurters<br>☐ Fish, steamed, grilled or baked<br>☐ Fish, fried (include take-away)<br>☐ Fish, tinned (salmon, tuna, sardines etc)	1. Never or less than once per month<br>2. 1–3 times per month<br>3. Once per week<br>4. 2–4 times per week<br>5. 5–6 times per week<br>6. Once per day<br>7. 2 or more times a day
Fruits	<u>Question:</u><br>Over the LAST 12 MONTHS, on average, how often did you eat the following fruits?<br><br>☐ Tinned or frozen fruit (any kind)<br>☐ Fruit juice<br>☐ Oranges or other citrus fruit<br>☐ Apples<br>☐ Pears<br>☐ Bananas<br>☐ Watermelon, rockmelon (cantaloupe), honeydew etc.<br>☐ Pineapple<br>☐ Strawberries<br>☐ Apricots<br>☐ Peaches or nectarines<br>☐ Mango or paw paw<br>☐ Avocado	1. Never or less than once per month<br>2. 1–3 times per month<br>3. Once per week<br>4. 2–4 times per week<br>5. 5–6 times per week<br>6. 6. Once per day<br>7. 2 or more times per day

Vegetables	<u>Question:</u><br>Over the LAST 12 MONTHS, on average, how often did you eat the following vegetables (including fresh, frozen and tinned)?<br><br>☐ Potatoes roasted or fried (i.e. hot chips)<br>☐ Potatoes cooked without fat<br>☐ Tomato sauce, tomato paste or dried tomatoes<br>☐ Fresh or tinned tomatoes<br>☐ Peppers (capsicum)<br>☐ Lettuce, endive, or other salad greens<br>☐ Cucumber<br>☐ Celery<br>☐ Beetroot<br>☐ Carrots<br>☐ Cabbage or Brussels sprouts<br>☐ Cauliflower<br>☐ Broccoli<br>☐ Silverbeet or spinach<br>☐ Green beans<br>☐ Bean sprouts or alfalfa sprouts<br>☐ Baked beans<br>☐ Soy beans, soy bean curd or tofu<br>☐ Other beans (include chick peas, lentils etc)<br>☐ Pumpkin<br>☐ Onion or leeks<br>☐ Garlic (not garlic tablets)<br>☐ Mushrooms<br>☐ Zucchini	1. Never or less than once per month<br>2. 1–3 times per month<br>3. Once per week<br>4. 2–4 times per week<br>5. 5–6 times per week<br>6. Once per day<br>7. 2 or more times per day
Beverage consumption	<u>Question:</u><br>Over the LAST 12 MONTHS, on average, how often did you drink the following?<br><br>☐ Cola drinks (not diet) (e.g. Coca-Cola®)<br>☐ Diet cola drinks (e.g. Diet Coke®)<br>☐ Other carbonated (e.g. fizzy/soft drinks)<br>☐ Cordials, fruit or sport drinks<br>☐ Milk or Soya Milk (including flavoured varieties)<br>☐ Fruit or vegetable juices<br>☐ Tea<br>☐ Herbal tea<br>☐ Coffee<br>☐ Water (including soda or plain mineral water)	1. Never<br>2. Less than 1–3 times per month<br>3. 1–3 times per week<br>4. 4–6 times per week<br>5. 1 time per day<br>6. 2 times per day<br>7. 3 or more times per day

Table S3. Foods included as part of the eight dietary components of the DASH diet.

Component	Included foods
**Fruits**	Fruit juice/Tinned fruit
	Orange
	Apple
	Pear
	Banana
	Melon
	Pineapple
	Strawberry
	Apricot
	Peach
	Mango
	Avocado
**Vegetables**	Tomato sauce
	Tomatoes
	Capsicum
	Lettuce
	Cucumber
	Celery
	Beetroot
	Carrots
	Cabbage
	Cauliflower
	Broccoli
	Spinach
	Peas
	Green beans
	Bean Sprouts
	Pumpkin
	Onion
	Garlic
	Mushrooms
	Zucchini
**Nuts and Legumes**	Nuts
	Baked beans
	Tofu
	Soy milk
	Peanut butter
	Other beans
**Grains**	All bran
	Weetbix
	Rice
	Pasta
	Vegemite
	Cornflakes
	Porridge
	Muesli
**Low fat dairy**	Skim milk
	Yoghurt
	Ricotta
	Cottage cheese
**Meat**	Beef
	Veal
	Lamb
	Pork
	Chicken
	Ham
	Salami

	Sausages
**Sodium**	Sodium
**Sweetened Beverages**	Sugar

Table S4. Individual food component intakes for participants in the current study compared to DASH diet recommendations. DASH scores are shown for each quintile.

Food Component	Q1<br>11-21	Q2<br>22-24	Q3<br>25-26	Q4<br>27-29	Q5<br>30-39	P-value	DASH diet recommendation*
Sodium (mg/day)	2 267	2 127	2 143	2 086	1 962	<0.001	< 2 500
Fruit (g/day)	119	167	210	254	330	<0.001	600 – 750
Vegetables (g/day)	99	106	117	125	142	<0.001	300 – 375
Grains (g/day)	166	187	210	226	267	<0.001	240 – 320 (bread)†<br>600 – 800 (pasta, porridge, rice)†
Nuts and/or legumes (g/day)	26	27	31	43	42	<0.001	17 – 21 (nuts)†<br>85 – 107 (legumes)†
Red and/or processed meat (g/day)	121	94	87	76	87	<0.001	<130
Dairy (g/day)	328	343	350	349	342	<0.001	100 – 200 (cheese)‡<br>500 – 1000 (milk)‡<br>400 – 800 (yoghurt)‡

Abbreviations: Q, Quintile

* Recommendations are from the RACGP DASH diet plan[12] and serving sizes are converted to g/day as per Australian Dietary Guidelines[36]

†This range provided only applies if this food is the sole source of that individual food component.

‡The DASH diet recommends low fat or non-fat dairy. This range provided only applies if this food is the sole source of that individual food component.
